# Supplementary figures and images for: Characterization of subtypes and transmitted drug resistance strains of HIV among Beijing residents between 2001-2016
Source: PLoS One. 2020 Mar 26;15(3):e0230779. doi: 10.1371/journal.pone.0230779 (PMC7098609; doi:10.1371/journal.pone.0230779)

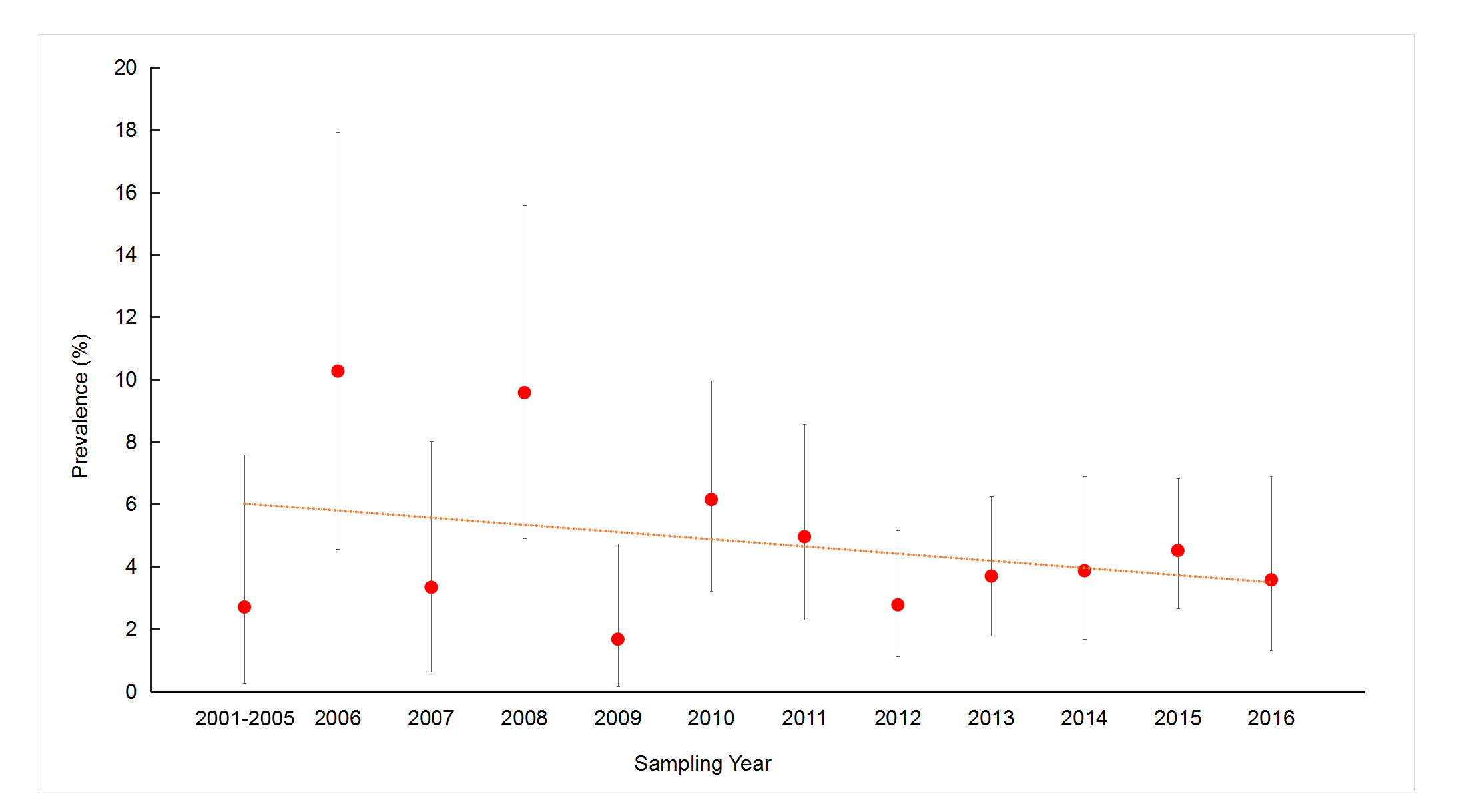

Supplement: S1 Fig — Vertical bars = 95% CI. The trend line is predicted overall prevalence of transmitted drug resistance. (TIF) [file pone.0230779.s001.tif]
