# Supplementary material for: Characterization of subtypes and transmitted drug resistance strains of HIV among Beijing residents between 2001-2016
Source: PLoS One. 2020 Mar 26;15(3):e0230779. doi: 10.1371/journal.pone.0230779 (PMC7098609; doi:10.1371/journal.pone.0230779)
Supplement: S1 Table — (DOCX) [file pone.0230779.s002.docx]

S1 Table. Demographic characteristics of individuals with genotype vs. those with non-genotype.

|  | Individuals with genotype (n=2,130) | Individuals with non-genotype (n=185) |
| --- | --- | --- |
| Sex |  |  |
| Men | 1979(92.9) | 169(91.4) |
| Women | 151(7.1) | 16(8.6) |
| Age at diagnosis(years)^a^ | 34(28-45) | 32(28-44) |
| CD4 counts (cells per μL)^b^ | 340(202-481) | 350(204-503) |
| Transmission risk group^c^ |  |  |
| Heterosexual | 567(27.2) | 50(28.9) |
| MSM | 1420(68.1) | 107(61.8) |
| IDU | 69(3.3) | 11(6.4) |
| Blood transfusion | 27(1.3) | 5(2.9) |
| Mother to child | 1(0.05) | 0(0) |
| Ethnicity |  |  |
| Han | 2049(96.2) | 175(94.6) |
| Minority | 81(3.8) | 10(5.4) |

Data are n (%) or median (IQR).

MSM=Men who have sex with men.

IDU=Injecting drug user.

^a^Data of genotype for n=2,125, Data of non-genotype for n=185.

^b^Data of genotype for n=1,858, Data of non-genotype for n=136.

^c^Data of genotype for n=2,084, Data of non-genotype for n=173.
