# Supplementary material for: Characterization of subtypes and transmitted drug resistance strains of HIV among Beijing residents between 2001-2016
Source: PLoS One. 2020 Mar 26;15(3):e0230779. doi: 10.1371/journal.pone.0230779 (PMC7098609; doi:10.1371/journal.pone.0230779)
Supplement: S2 Table — (DOCX) [file pone.0230779.s003.docx]

S2 Table.Univariable logistic regression analysis of CD4 counts associated with transmitted drug resistance with multiple imputation.

|  | odds ratio (95% CI) | p value |
| --- | --- | --- |
| CD4 counts (cells per μL) |  |  |
| <200 | Reference |  |
| 200-349 | 1.05(0.57-1.95) | 0.82 |
| 350-499 | 0.93(0.48-1.79) | 0.71 |
| >499 | 1.28(0.69-2.40) | 0.49 |
