# Supplementary material for: Characterization of subtypes and transmitted drug resistance strains of HIV among Beijing residents between 2001-2016
Source: PLoS One. 2020 Mar 26;15(3):e0230779. doi: 10.1371/journal.pone.0230779 (PMC7098609; doi:10.1371/journal.pone.0230779)
Supplement: S4 Table — (DOCX) [file pone.0230779.s005.docx]

S4 Table. Sensitivity analysis of CD4 counts associated with transmitted drug resistance by excluding sampling phase 2009-2011.

|  | Univariable logistic regression analysis |  |
| --- | --- | --- |
|  | odds ratio (95% CI) | p value |
| CD4 counts (cells per μL)a |  |  |
| <200 | Reference |  |
| 200-349 | 0.96(0.46-1.98) | 0.9 |
| 350-499 | 1.10(0.52-2.28) | 0.82 |
| >499 | 1.37(0.69-2.79) | 0.37 |

aData for n=1,458.
