# Supplementary material for: Characterization of subtypes and transmitted drug resistance strains of HIV among Beijing residents between 2001-2016
Source: PLoS One. 2020 Mar 26;15(3):e0230779. doi: 10.1371/journal.pone.0230779 (PMC7098609; doi:10.1371/journal.pone.0230779)
Supplement: S5 Table — (DOCX) [file pone.0230779.s006.docx]

S5 Table. Sensitivity analysis of CD4 counts associated with transmitted drug resistance by excluding sampling phase 2012-2014.

|  | Univariable logistic regression analysis |  |
| --- | --- | --- |
|  | odds ratio (95% CI) | p value |
| CD4 counts (cells per μL)a |  |  |
| <200 | Reference |  |
| 200-349 | 1.16(0.58-2.32) | 0.68 |
| 350-499 | 0.55(0.22-1.28) | 0.18 |
| >499 | 1.50(0.72-3.11) | 0.28 |

aData for n=1,143.
