# Supplementary material for: Characterization of subtypes and transmitted drug resistance strains of HIV among Beijing residents between 2001-2016
Source: PLoS One. 2020 Mar 26;15(3):e0230779. doi: 10.1371/journal.pone.0230779 (PMC7098609; doi:10.1371/journal.pone.0230779)
Supplement: Attachment — Submitted filename: renamed_0442e.docx [file pone.0230779.s009.docx]

**Response to reviewers**

PONE-D-19-15622R1
Characterization of subtypes and transmitted drug resistance strains of HIV among Beijing residents between 2001-2016
PLOS ONE

**Dear Editor and Reviewers:**

We would like to thank *Plos One* for giving use the opportunity torevise our manuscript. We thank the reviewer for this pertinent and insightful comment. We have carefully taken their comments into consideration in preparing our revision. Below is our response to their comments.

Reviewer #2: The revised manuscript is substantially improved; however, one issue remains pertaining missing data.

The revised methods section states multiple imputation was used to handle missing data. However, the footnotes of the tables indicate differential sample sizes for certain variables. For instance, there were a total of 2130 sequences, but the number of sequences for each stratum of CD4 count interval in Table 3 only sums to 1858 (473+519+455+411). Further, the discussion states, "When missing, the CD4 counts was imputed, but imputation was only required for 454 12.8% of the study population." Did the authors only account for imputation in the regression analyses but not the descriptive data? If so, this is not standard practice and should be explained.

Reply:

As discussed in this our previous response (see above), we used listwise deletion method to handle the missing data. We also attempted to use the more powerful method-multiple imputation. The introduction of multiple imputations added more problems in the analysis. As kindly mentioned by the reviewer, the imputation in the regression analysis is not a more conventional and widely used standard practice. This is now dropped in our final analysis.

The data missing in our cohort mainly occurred for age (0.2%), transmission risk group (2.2%) and CD4 counts (12.8%). We think the effect of data missing in the first two variables on the association were negligible, therefore we mainly focus on the data missing in CD4 counts. Referring on the literature on how to handle the problem of miss data, at least ten HIV TDR articles published in Lancet infectious disease, Lancet HIV, CID, JID, AIDS, and Plos One within the past ten years were used to guide our analysis. Of the ten papers, only one in JID used listwise method to handle miss data[1]. The remaining nine articles did not mention what method was used to handle miss data[2-10]. Since the most popular statistic software used listwise deletion to handle miss data by default, we have assumed that the listwise method was used. On the contrary, multiple imputation was never mentioned at all.

Therefore, we decide to use listwise method rather the multiple imputation to handle the missing data throughout the article. Nevertheless, we did also try multiple imputation to handle CD4 missing in logistics analysis(S2 Table). The results from listwise and multiple imputation were consistent.

Moreover, when four sensitivity experiments were carried out by excluding individual sampling phase(S3-6 Table, the association between CD4 and TDR was still not significant.

If the missing data were assumed to be missing at random and multiple imputation was truly employed to handle missing data, the methods section should provide more details on how and why multiple imputation was conducted. What variables had missing data and what percentage? What variables had missing data imputed? What variables were considered in the imputation model? How many imputed data sets were used?

Reply:

Age (0.2%), transmission risk group (2.2%) and CD4 counts (12.8%) had missing data. CD4 counts had missing data imputed. CD4 variable was considered in the imputation model. Five imputed data sets were used.

The program for multiple imputation in R was list below:

library(lattice)

library(MASS)

library(nnet)

library(mice)

imp<-mice(data,m)

fit<-with(imp,analysis)

pooled<-pool(fit)

summary(pooled)

References

1 Wan-Lin Yang,Roger Kouyos,Alexandra U. Scherrer,et al.Assessing the paradox between transmitted and acquired HIV type 1 drug resistance mutations in the Swiss HIV cohort study from 1998 to 2012.J Infect Dis 2015;212:28-38.

2 L Marije Hofstra,Nicolas Sauvageot,Jan Albert,et al.Transmission of HIV drug resistance and the predicted effect on current first-line regimens in Europe.Clin Infect Dis 2016;62:655-663.

3 Santiago ávila-Ríos, Claudia García-Morales, Margarita Matías-Florentino,et al. Pretreatment HIV-drug resistance in Mexico and its impact on the effectiveness of first-line antiretroviral therapy:a nationally representative 2015 WHO survey,Lancet HIV 2016;3:e579-e591.

4 Theppharit Panichsillapakit,Davey M. Smith,Joe O. Wertheim,et al.Prevalence of transmitted HIV drug resistance among recently infected persons in San Diego, CA 1996-2013.J Acquir Immune Defic Syndr 2016;71:228-236.

5 Junko Hattori,Teiichiro Shiino,Hiroyuki Gatanaga, et al.Characteristics of transmitted drug-resistant HIV-1 in recently infected treatment-naive patients in Japan.J Acquir Immune Defic Syndr 2016;71:367-373.

6 Patrycja Machnowska, Karolin Meixenberger, Daniel Schmidt, et al. (2019) Prevalence andpersistence of transmitted drug resistancemutations in the German HIV-1 SeroconverterStudy Cohort. PLoS One2019; 14:e0209605.

7 Raph L. Hamers, Carole L. Wallis, Cissy Kityo,et al.HIV-1 drug resistance in antiretroviral-naive individuals in sub-Saharan Africa after roll-out of antiretroviral therapy:a multicentre observational study. Lancet Infect Dis 2011;11:750-759.

8 Donn J. Colby,Trevor A. Crowell,Sunee Sirivichayakul, et al.Declining trend in transmitted drug resistance detected in a prospective cohort study of acute HIV infection in Bangkok, Thailand.J Int AIDS Soc 2016;19:20966.

9 Junko Tanuma, Vo Minh Quang,Atsuko Hachiya, et al.Low prevalence of transmitted drug resistance of HIV-1 during 2008-2012 antiretroviral therapy scaling up in Southern Vietnam.J Acquir Immune Defic Syndr2014;66:358-364.

10 Vercauteren J , Wensing A , Vijver D V D , et al. Transmission of drug-resistant HIV-1 is stabilizing in Europe. Journal of Infectious Diseases, 2009, 200(10):1503-1508.

#2.  Also ensure that they manuscript has been reviewed thoroughly by a native English speaker and/or professional editing service.

Yes, we did.

We hope that our revision is acceptable, and we look forward to hearing from you soon.
