# Supplementary material for: Characterization of subtypes and transmitted drug resistance strains of HIV among Beijing residents between 2001-2016
Source: PLoS One. 2020 Mar 26;15(3):e0230779. doi: 10.1371/journal.pone.0230779 (PMC7098609; doi:10.1371/journal.pone.0230779)
Supplement: Attachment — Submitted filename: plos response.docx [file pone.0230779.s010.docx]

**Response to reviewers**

PONE-D-19-15622R1
Characterization of subtypes and transmitted drug resistance strains of HIV among Beijing residents between 2001-2016
PLOS ONE

**Dear Editor and Reviewers:**

We would like to thank *Plos One* for giving use the opportunity to revise our manuscript. We thank the reviewer for this pertinent and insightful comment. We have carefully taken their comments into consideration in preparing our revision. Below is our response to their comments.

Two minor comments:

1. Methods (page 9, Line 175): Please revise the sentence, "However when CD4 data were missing the multiple imputation was applied to perform logistics analysis." to read: "However, since 12.8% of data were missing for CD4 count, a sensitivity analysis was performed using multiple imputation to handle missing data (m=5)."

Yes, we did it. This sentence is far more accurate than the former one.

2. Please check p values in Table 3. The adjusted odds ratio for women was 0.26 (0.04-0.85) but the p value is >0.05 (i.e., p=0.06). The p value for this effect size and CL limit <1 would be expected to have a p value <0.05.

Thank you for your serious and careful proofreading. As the first author, though I have read the manuscript for more than 20 times, I still did not notice this inconsistence in Table 3. Honestly, when I first got this comment, my hair stood on end. Because this inconsistence could mean that either the OR or the p value is wrong. If it is true, the overall result of logistic analysis could be overthrew. That will be a nightmare for me. Fortunately, when I double checked the result of logistic analysis, I confirmed that there is no wrong in our logistic analysis. Therefore, the final question could be summed up as "Should the CI of OR always be consistent with the p value?" This question sounds like unprofessional. But it is the truth under our circumstance. Why is that so? We speculated that the sample size of women is too small to reach significance. The number of women in our study is trivial compared with the number of men( 151 vs 1979). Our recent national survey with larger sample size of women confirms that our speculation is correct. Moreover, the Chi-squared test also showed that the difference is non-significant(x-squared=3.0, p value=0.08).

Finally, we will like to express my appreciation for all these insightful comments and hard work in this reviewing. I learnt a lot of statistical knowledge in the submission. Yes, the process of submission is also the process of learning.

We hope that our revision is acceptable, and we look forward to hearing from you soon.
